# An algorithmic strategy for measuring police presence with GPS data

**DOI:** 10.1186/s40163-024-00221-x

**Published:** 2024-08-24

**Authors:** Robin Khalfa, Thom Snaphaan, Wim Hardyns

**Affiliations:** 1 Department of Criminology, Criminal Law and Social Law Ghent University, Universiteitstraat 4, 9000 Ghent, Belgium; 2 Centre of Expertise Safe and Resilient Society, Avans University of Applied Sciences, Hugo de Grootlaan 37, 5223 LB ‘s-Hertogenbosch, Netherlands; 3 Faculty of Social Sciences, University of Antwerp, Sint-Jacobstraat 2, 2000 Antwerp, Belgium

**Keywords:** Big data, Crime hot spots, Focused deterrence, GPS data, Police patrols, Police presence, Track and trace

## Abstract

This study introduces an algorithmic strategy for measuring dimensions of police presence at microgeographic units using GPS data from police patrol units. The proposed strategy builds upon the integrated theory of hot spots patrol strategy from Sherman et al. (Journal of Contemporary Criminal Justice 30:95–122, 2014), focusing on three key dimensions: the frequency, duration, and intermittency of police presence. This study provides pseudocodes for the algorithm, facilitating the pre-processing of GPS-derived data sequences to generate measures of these three central concepts. The measures presented in this article offer a framework for investigating the impact of police presence on crime and other relevant crime-related outcomes at microgeographic units, using GPS data. This algorithmic strategy may further contribute to the development of evidence-based strategies in place-based policing initiatives.

## Introduction

Hot spots policing, which concentrates police resources on high crime microgeographic units or crime hot spots, has proven to be an effective crime reduction strategy (Braga et al., 2019; Ratcliffe, 2016; Sherman & Weisburd, 1995). In this regard, hot spots policing strategies are mainly predicated on the assumption that (targeted) police presence in crime hot spots will deter offenders and reduce crime, both on the short and long term (Sherman et al., 2014). However, to date, a critical gap in scientific literature remains regarding the operationalization and measurement of police presence in crime hot spots.

With regard to the operationalization of police presence in crime hot spots, however, Sherman et al., (2014) proposed an ‘integrated theory of hot spots patrol strategy’. Although this theory presents a broader framework for establishing hot spots policing strategies, it identifies three key quantifiable dimensions that should be considered when defining police presence in the context of hot spots policing strategies: (1) frequency, the number of times police units visit a hot spot in a given period; (2) duration, the amount of time police units spend in a hot spot during each visit; and (3) intermittency, the time in between police departures and arrivals in hot spots. These three dimensions are assumed to be pivotal in establishing deterrent effects and hence, reduce crime in hot spots.

While these three dimensions may provide a clearer operationalization for defining (quantifiable) dimensions of police presence in crime hot spots, questions remain on how they can be measured more efficiently and accurately and, hence, how studies can use measurements of these dimensions to evaluate hot spots policing strategies. Traditionally, studies evaluating the effects of police presence on crime in hot spots have relied on more expensive and laborious strategies to measure police presence, such as the use of student observers to estimate the time spent by officers in crime hot spots. Nowadays, with the advent of Global Positioning System (GPS)-monitored police systems, researchers are able to collect more granular, accurate and cost-effective data of police activity. In this regard, GPS data provide a detailed record of officers’ movements, allowing for the precise measurement of the distribution of police presence.

The present article proposes an algorithmic strategy that facilitates the measurement of the frequency, duration and intermittency of police presence in microgeographic units using (big) data derived from GPS-monitored police systems (e.g., track and trace systems of police patrol cars). This approach aims to facilitate and enhance the production of scientific knowledge regarding the nuanced dynamics surrounding overall police presence in microgeographic units, providing valuable insights for refining and optimizing hot spot policing strategies and place-based policing strategies in general.

## Three dimensions of police presence in high crime hot spots: measuring frequency, duration, and intermittency

At the heart of Sherman and colleagues’ (2014) integrated theory lies the assumption that the deterrent effect of police presence on criminal behaviour is mediated by the perceived risk of apprehension. This assertion highlights the critical role of perceived risk in shaping offenders' decision-making processes and deterring criminal activity (Bucci, 2024). While thus not introducing new assumptions, Sherman and colleagues underscore an often-overlooked aspect in existing hot spots policing strategies, namely the study of the specific dimensions and conditions under which police presence can deter and reduce crime in these areas. Sherman and colleagues, among several others (Ariel et al., 2016; Dau et al., 2023; Williams & Coupe, 2017), thereby recognize the potential of data derived from GPS-monitored devices to enhance measures of the overall police presence in microgeographic units, enabling more precise and fine-grained testing of specific propositions regarding the effects of targeted police presence on crime at microgeographic units. It is crucial to acknowledge, however, that the measurement of police presence across the three aforementioned dimensions (frequency, duration, and intermittency) requires careful consideration of specific methodological choices and aspects.

First of all, it is important to determine whether to measure (and evaluate) police presence across police units or for each unit separately. Two distinct approaches thus address this question: overall and unit-by-unit measurement of police presence. Overall measurement involves aggregating the combined activity of all patrol units within a defined spatiotemporal frame, offering a broader overview of overall police presence in that area. Unit-by-unit measurement, on the other hand, involves—as the name implies—measuring police presence on a unit-by-unit basis, allowing for the examination of the activities of each patrol unit individually within a specific police district. In this article, we primarily focus on overall measurement, providing an algorithmic strategy on how the frequency, duration, and intermittency of police presence can be measured and assessed at an aggregated level, i.e., across police patrol units.^1^ The overall measurement approach holds particular significance when evaluating place-based policing strategies, where the primary interest usually lies in estimating the total (overall) deterrent and crime reduction effects stemming from police presence in high-crime areas. In this regard, however, it is worth mentioning that the aim of the algorithmic strategy proposed is not to measure specific types or forms of targeted police presence, such as police vehicle stops (see Dau, 2023), but to facilitate the measurement of overall levels of police presence in terms of the three aforementioned dimensions.

Furthermore, as Hutt et al., (2021) emphasize, the accuracy of the employed GPS data warrants careful consideration. Specifically, apart from the systematic bias that may exist within GPS location measurements (e.g., signal multipath, satellite orbital errors, clock bias etc., for a more comprehensive overview see Hutt et al., 2021), the temporal resolution (i.e., refresh rate) at which GPS pings are recorded along patrol unit paths may significantly impact the measurement of police presence in micro-places. Hence, depending on the frequency with which GPS pings are recorded, measurement error regarding the locations in a police unit’s path or route is introduced. The latter especially warrants consideration when GPS data are characterized by slower refresh rates, which is often the case for foot patrol data. Hutt et al., (2021) therefore argue that the paths or routes taken between consecutive GPS pings require interpolation. This entails the estimation of patrol trajectories between known GPS data points, for example by applying a ‘join-the-dots’ approach whereby a police unit’s path is assumed to be a straight line between GPS pings, holding the speed between consecutive pings constant. Although such interpolation strategies may prove valuable and result in more accurate measurements and representations of police presence in micro-places, the computational burden imposed can be substantial, particularly when dealing with large datasets comprising a large number of GPS data points. In addition, interpolation may become even more complex when one aims to measure police presence across individual police units (i.e., collective measurement) rather than aiming to measure police presence for each unit individually. In this regard, interpolation may go beyond what is practical for police organisations or researchers to compute or use. The algorithmic strategy proposed in this article therefore introduces a more simplistic approach by counting GPS pings within a defined microgeographic unit based on a specific temporal threshold value (e.g., GPS refresh rate) (Ariel et al., 2016; Williams & Coupe, 2017). While we acknowledge that this approach may be less suitable for GPS datasets characterised by slower refresh rates, it may offer a readily usable tool for police organisations and researchers with limited computational resources using fine-grained spatiotemporal GPS data of police activity, providing easily understandable measures of police presence.

### Measuring instances of police presence along GPS sequences

**Fig. 1:**
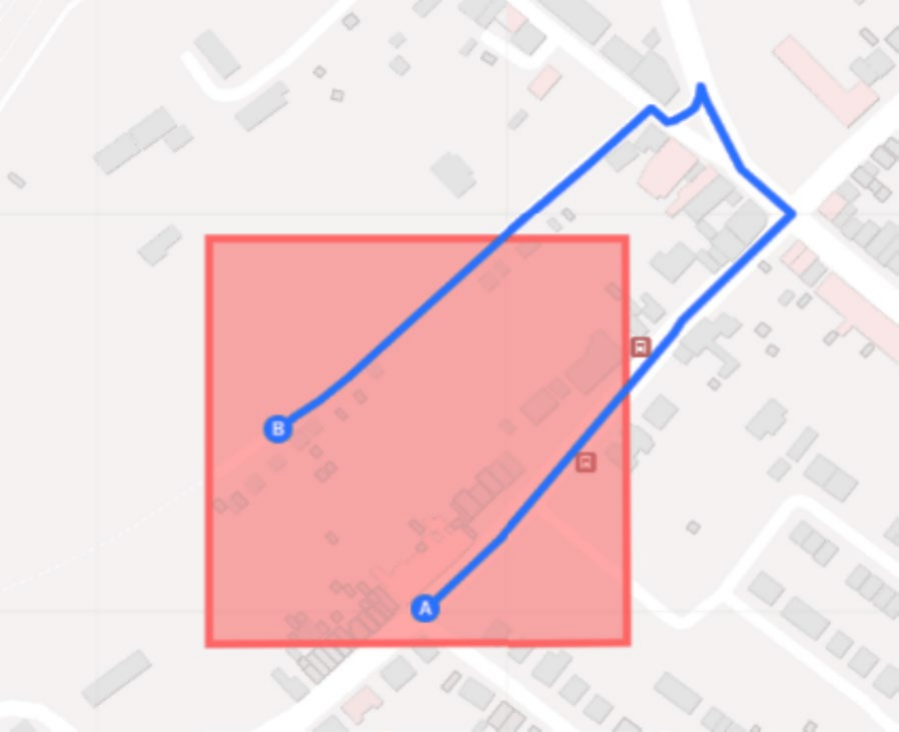
Example of a situation where police patrol units have to exit the crime hot spot to be able to patrol the entire hot spot

In contrast to interpolation methods that approximate patrol trajectories based on GPS signal intervals, the algorithmic strategy proposed involves setting a temporal threshold to signify instances of police presence within a defined micro-place over a specified time period. This threshold, which can be for example set according to the GPS refresh rate, allows for counting unique GPS pings within a specific microgeographic unit. Faster refresh rates lead to more accurate and representative counts, as outlined before. For example, suppose a GPS device records patrol positions every 4 s, and that this 4 s interval serves as the temporal threshold. The algorithm then organizes unique GPS pings in ascending order, grouped by the defined micro-geographic units. Using the 4 s threshold, the algorithm generates a data frame with incremented counter values whenever the time difference between two unique timestamps exceeds the set threshold (e.g., 4 s), signifying separate instances of police presence. This can be translated into the following pseudocode which forms the basis for calculating the frequency, duration, and intermittency of police presence later on:
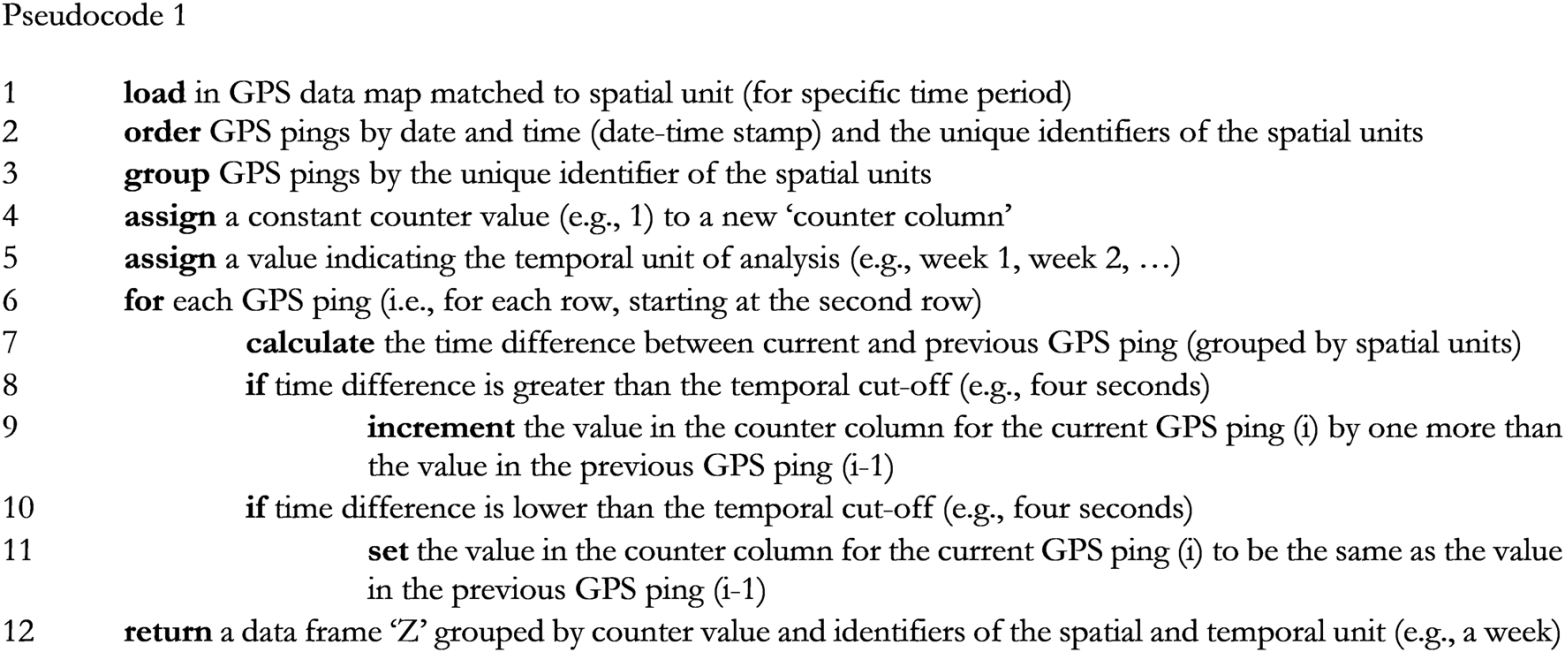


An important consideration here is that in certain situations, however, police patrol units may not be able to fully patrol a microgeographic unit or hot spot without exiting the area, potentially causing disruptions in how one wants to measure and capture police presence within that specific area. Figure 1 provides an example of such a situation. Consequently, researchers or police organisations are faced with a decision regarding whether to mitigate or disregard these potential disruptions. However, for those opting to address this concern, two overarching strategies emerge as viable solutions. One strategy involves creating a buffer zone around the microgeographic units and spatially linking GPS data points to the buffered units based on the principle of the largest overlap. This approach allows for a more comprehensive representation of police presence within the microgeographic unit, accounting for instances where patrols extend slightly beyond the defined boundaries. Another strategy is to increase the temporal cut-off value used in the algorithm to define instances of police presence. For example, instead of using the refresh rate as the temporal threshold (e.g., 4 s), a higher temporal threshold (e.g., 30 s) can be set, providing a temporal rather than a spatial buffer. This adjustment accommodates situations where police officers temporarily leave the hot spot and subsequently return to patrol the remaining area, ensuring that their absence does not result in separate instances being incorrectly identified. By implementing these strategies, the measurement of police presence in microgeographic units can be more accurately represented, considering potential interruptions and optimizing the understanding of police activities within hot spots. Both strategies can be easily implemented in the provided algorithm, by map matching the data to microgeographic units including spatial buffers of the microgeographic units or by incrementing the temporal threshold.

### Capturing the frequency, duration and intermittency of police presence

The frequency of police presence refers to the total number of times police patrol units are present or visit a specific area, such as a hot spot, within a defined time period (e.g., one day, one night, one week etc.) (Koper, 1995; Mitchell, 2017; Sherman et al., 2014; Williams & Coupe, 2017). The underlying theoretical assumption is that the frequency of police presence in targeted micro-places can influence the local deterrent effects of police presence on crime reduction in those areas (Koper, 1995; Sherman et al., 2014). Therefore, it is proposed that a higher frequency of police presence in each crime hot spot, achieved through foot or car patrols, enhances both initial and residual deterrence. This increase in frequency is expected to result in a drop in crime following police presence in a crime hot spot, as well as sustained crime reduction over time.

Furthermore, while Sherman and colleagues generally write about dosage, we prefer to talk about the duration of police presence to refer to the total time of police presence in a microgeographic unit or hot spot per police visit. Hence, dosage is defined as the combination of both the frequency and duration of police presence. According to Koper's (1995) research, the ideal length of police presence is between 11 and 15 min per police visit. Longer police presence in crime hot spots can lead to higher levels of initial and residual deterrence, until the point where the effectiveness of deterrence begins to decay, typically after 15 min of police presence. Nevertheless, recent studies have indicated that even shorter police visits (e.g., police vehicle stops of 2 to 5 min and 6 to 10 min in the hottest spots) may yield positive results (Dau, 2023; Dewinter, 2023). The advent of GPS-monitored police systems provides increased opportunities to examine the relationship between different levels of police presence duration in crime hot spots and the subsequent reduction in crime frequencies within those areas. Additionally, it enables investigation into the precise timing when deterrence decay occurs.

In addition, it is assumed that police presence should be unpredictable and that potential offenders should be uncertain about police departures and arrivals in crime hot spots. Therefore, it is suggested that a greater variability in the intermittency of police presence positively affects the unpredictability of ‘‘when police will next appear in a hot spot at any given time’’ (Sherman et al., 2014, p. 105). This, in turn, can impact the uncertainty offenders face regarding their risk of apprehension. Thus, the concept of intermittency primarily pertains to the time intervals between police departures and arrivals in crime hot spots. In this regard, it is theoretically assumed that when the intermittency of police presence exhibits higher variance in hot spots, this will enhance the unpredictability and uncertainty surrounding police activities in those areas, potentially leading to reduced frequency and severity of crime.

Based upon the pseudocode of the algorithm that was presented to create a data frame ‘Z’ that indicates police visits in micro-places with incremented counter values, we propose the following pseudocode to create separate measures of the frequency, duration and intermittency of police presence in micro-places (e.g., on a weekly basis):
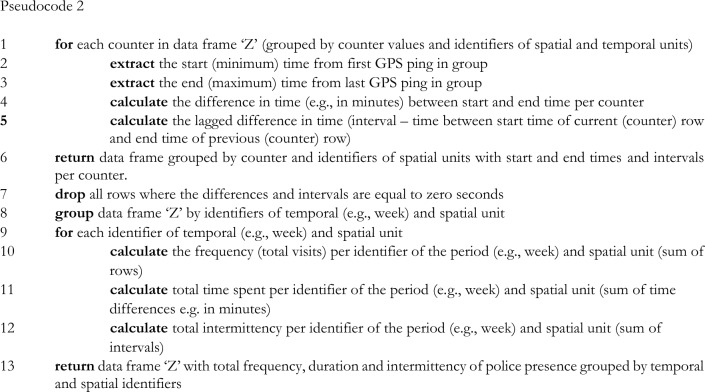


This algorithm enables defining the start and end times of instances of police presence in microgeographic units and can be used to measure the frequency, duration and intermittency of police visits in each microgeographic unit. The frequency of police presence is simply the sum of the number of recurring police visits during a specific period in time (e.g., one week) in a microgeographic unit. The total duration of police presence is the sum of the differences in time (e.g., seconds) between all the start and end times of all police visits in one microgeographic unit for a specified period in time.^2^ The intermittency of police presence is the difference in time between a police departure and a new police arrival for each sequence of police presence in each microgeographic unit. This measure is simply based on the difference in time between the end of a time interval and the beginning of a new time interval (lagged value) across police visits. However, instead of summing up these intervals to a total measure of the intermittency of police presence, Sherman et al. (2014) argue that it would be more meaningful to calculate the variance of the intermittency of police presence in each separate microgeographic unit over a given time period (e.g., one week), as it is assumed that a higher variance will result in higher levels of local deterrence and will eventually lead to lower crime frequencies.

## Discussion and conclusion

This article proposes an algorithmic strategy to measure three central dimensions of police presence at small spatiotemporal scales using GPS data: (1) frequency, (2) duration, and (3) intermittency of police presence. The first algorithm enables the identification of separate instances of police presence along sequences of GPS datapoints, while the second algorithm creates separate measures of police presence for each microgeographic unit. These algorithms can be combined into an integrated algorithm that executes these different tasks sequentially.

Overall, the operationalisations of the measures presented in this study can serve as a means to examine the impacts of these dimensions of police presence on crime counts or other pertinent crime-related outcome measures at microgeographic units, utilizing GPS data. In that regard, it would be interesting to examine whether the proposed theoretical propositions of the effects of police presence on crime actually align with empirical measures and how these measures behave across different contexts and spatiotemporal units of analysis. The measures can for example be used to estimate optimal dosage-response curves in light of future experimental research of place-based policing interventions (e.g., place-based predictive policing), in which the frequency, duration and intermittency of police presence in experimental treatment groups can be plotted against the backdrop of the dimensions of police presence used in control groups. However, measuring dimensions of police presence entails acknowledging and accounting for the limitations of data that are derived from GPS-monitored systems and the strategy that is used to analyse the effects of police presence on crime at microgeographic units.

As outlined before, the algorithmic strategy proposed in this article relies on counting unique GPS pings within microgeographic units to signify instances of police presence, allowing for the creation of separate measures of the frequency, duration, and intermittency of police presence. The proposed algorithm may therefore provide less accurate measures when the GPS data employed comprises slower refresh rates. We acknowledge this specific limitation, which may affect estimations of the deterrence of police presence at microgeographic units. However, while interpolating the presumed trajectories between successive GPS signals may offer enhanced accuracy in this context, it concurrently imposes considerable computational demands, rendering it less pragmatic for researchers and practitioners dealing with large GPS datasets. In light of increasing digitization and datafication, this concern regarding computational efficiency and practicality may assume even greater significance.

Moreover, it is imperative to consider the implications for police presence measurement when patrol units encounter constraints preventing continuous coverage of microgeographic units or hot spots without exiting the area. To address this issue, two broad strategies have been proposed in this article: the incorporation of a spatial buffer zone or a temporal buffer. These buffers aim to accommodate instances where police officers temporarily leave the hot spot and subsequently return to resume patrol, ensuring that such absences do not lead to the erroneous identification of separate patrol instances. However, while these strategies offer potential value in certain contexts, it is essential to recognize their applicability primarily to situations involving distinct, isolated areas or hot spots. Challenges emerge when contiguous hot spots exist or when the objective is to measure police presence across all microgeographic units encompassing the entire study area. In such scenarios, the utilization of spatial or temporal buffers may pose difficulties. A potential approach entails the consideration of double-counting police presence by including it for all adjacent areas of interest. This approach not only addresses instances where police patrols traverse the boundaries of two microgeographic units but also aligns with the objective of capturing what offenders see or perceive, rather than solely focusing on the location of police units themselves.

Another important consideration is that there may be differences in the measurement of police presence according to the microgeographic units used in empirical studies. For example, there may be important differences in how police resources are distributed within grid cells when compared to street segments. The question is also whether police presence should be captured collectively, across all police patrol units within one police district or individually for each police patrol unit separately and how measures of the effects of these quantitative dimensions of police presence should be evaluated in light of qualitative dimensions of police presence, such as police legitimacy. Regarding the latter, it is thus not only important to consider when and for how long police officers should be present in certain places, but also to think about ‘what the police should do at micro-places or crime hot spots’. Some places might benefit from more community-oriented or problem-solving approaches, instead of traditional law enforcement tactics such as stop-and-frisks (Braga et al., 2019). These are all questions that can be addressed in future research, especially in evaluation studies, which should focus on tailoring these operationalisations to different contexts and practices, allowing for a more nuanced understanding of the subject matter.

## Data Availability

Not applicable.

## Footnotes

1 However, the algorithmic strategy proposed may be easily adapted to the individual measurement of police presence (e.g., a patrol car unit) by grouping the data accordingly.

2 Although we present the duration of police presence as a sum, one may calculate the mean of the total time police patrol units were present in one specific micro-place by dividing the total time by the total number of visits in that micro-place in any given time period (e.g., one week).
